# Real time flow imaging in atrial fibrillation

**DOI:** 10.1186/1532-429X-17-S1-Q25

**Published:** 2015-02-03

**Authors:** Michael Markl, Daniel C Lee, Maria L Carr, Jason Ng, Ning Jin, Alex J Barker, James C Carr, Jeffrey J Goldberger

**Affiliations:** 1 Northwestern University, Chicago, IL, USA; 2 Siemens, Columbus, OH, USA

## Background

Atrial fibrillation (AF) is a common cardiac arrhythmia, affecting 33.5 million patients worldwide. The most serious complication is stroke, which is generally attributed to embolism of thrombus from the left atrium (LA). Studies utilizing transesophageal echocardiography (TEE) have shown that decreased blood flow velocities in the LA are risk factors for stroke in AF^1,2^. However, TEE requires esophageal intubation and cannot fully assess the complex 3D pattern of flow in the LA. In addition, a systematic analysis of the impact of beat-to-beat variations on LA flow characteristics and thus thromboembolic risk is lacking, particularly in the presence of arrhythmia. To better elucidate the role of beat-to-beat flow variations in different AF phenotypes (arrhythmia vs. sinus) we utilize a recently introduced real-time 2D flow imaging method which has shown promising results^3^. The goal of this feasibility study was to investigate different measures of LA flow to better understand the impact of different AF phenotypes on LA hemodynamics.

## Methods

In 7 AF patients (5 with arrhythmia, 2 in sinus rhythm), highly accelerated real-time 2D flow imaging (echo planar readout, shared velocity ending spatio-temporal parallel acceleration) with through plane velocity encoding (venc=80-150cm/s) was performed. Images were acquired during breath-holding in 2D planes along the LA short axis to investigate the impact of irregular heart rate in AF on LA flow and velocities (total scan time=10 seconds). To achieve sufficiently high frame rates needed for real-time flow acquisitions (45-50ms), the 2D flow imaging pulse sequence combines an echo planar imaging (EPI) readout module and with parallel acceleration in the temporal direction (T-PAT) and a novel reconstruction algorithm, shared velocity encoding^3^. Data analysis included the calculation of LA flow-time and mean velocities-time curves in a region of interests delineating the LA boundary (see figure).

## Results

**Figure 1:**
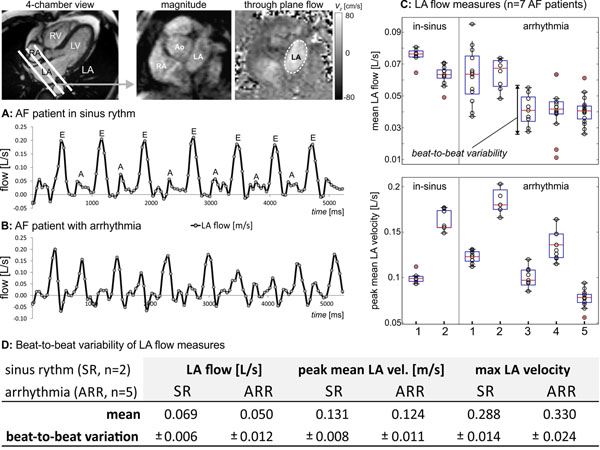
Real-time flow imaging in AF in a patient in sinus rhythm (A) and a subject with arrhythmia (B) and corresponding real-time flow curves at the mid-atrial short axis level over multiple heart beats (frame rate=50ms). C: Beat-to-beat variability of mean LA flow and peak mean LA velocity for all 7 patients included in the study. D: Quantification of mean and beat-to-beat variability of LA flow parameters.

As shown in the figure, real-time flow imaging could reliably assess LA flow variations over multiple heart beats for both sinus-rhythm and arrhythmia. Quantification of average and beat-to-beat variability of mean LA flow, peak mean LA velocities, and maximum LA through plane velocities (Fig 1C, D) clearly demonstrated elevated beat-to-beat variability in arrhythmia compared to sinus rhythm for all analyzed LA flow measures.

## Conclusions

The findings of this study demonstrate the feasibility of real-time flow imaging to measure beat-to-beat flow variation in AF patients with arrhythmia and compared to sinus rhythm. Noticeably, there was high inter-individual variability in peak LA velocities and their beat-to-beat variability indicating the potential of real-time flow MRI to identify individual patients with altered AF induced flow characteristics.

## Funding

AHA (12GRNT12080032) and NIH-NHLBI (1R21HL113895).

